# Development of machine-learning-driven signatures for diagnosing and monitoring therapeutic response in major depressive disorder using integrated immune cell profiles and plasma cytokines

**DOI:** 10.7150/thno.102602

**Published:** 2024-10-28

**Authors:** Shen He, Faming Zhao, Guangqiang Sun, Yue Shi, Tianlun Xu, Yu Zhang, Siyuan Li, Linna Zhang, Xingkun Chu, Chen Du, Dabing Yang, Jing Zhang, Changrong Ge, Jingjing Huang, Zuoquan Xie, Huafang Li

**Affiliations:** 1 Department of Psychiatry, Shanghai mental health center, Shanghai Jiao Tong University School of Medicine, Shanghai, China.; 2 Key Laboratory of Environmental Health, Ministry of Education & Ministry of Environmental Protection, School of Public Health, Tongji Medical College, Huazhong University of Science and Technology, Wuhan, China.; 3 State Key Laboratory of Drug Research, Shanghai Institute of Materia Medica, Chinese Academy of Sciences, Shanghai 201203, China.; 4 Department of Psychiatry, The Second Affiliated Hospital of Xinxiang Medical University, Xinxiang, Henan 453002, China.; 5 Green Valley Pharmaceutical Technology Co., Ltd., Shanghai 201203, China.; 6 Clinical Research Center for Mental Disorders, Shanghai Pudong New Area Mental Health Center, School of Medicine, Tongji University, Shanghai, China.; 7 Shandong Laboratory of Yantai Drug Discovery, Bohai Rim Advanced Research Institute for Drug Discovery, Yantai, Shandong 264117, China.; 8 Shanghai Key Laboratory of Psychotic Disorders, Shanghai, China.

**Keywords:** major depressive disorder, immune cell, CyTOF, cytokines, machine-learning, biomarkers

## Abstract

**Background:** Diagnosis and treatment efficacy of major depressive disorder (MDD) currently lack stable and reliable biomarkers. Previous research has suggested a potential association between immune cells, cytokines, and the pathophysiology and treatment of MDD.

**Objective:** This study aims to investigate the relationship between immune cells, cytokines, and the diagnosis of MDD and treatment response, further utilizing machine learning algorithms to develop robust diagnostic and treatment response prediction models.

**Methods:** Using mass cytometry by time-of-flight (CyTOF) technology and high-throughput cytokine detection, we analyzed 63 types of immune cells from 134 pre-treatment MDD patients. Among these patients, plasma data for 440 cytokines were obtained from 84 individuals. Additionally, we conducted the same set of immune cell and cytokine analyses on 50 healthy controls (HC). An 8-week follow-up was conducted to observe post-treatment changes in immune cells and cytokines.

**Results:** By combing eight machine-learning algorithms with CyTOF and cytokine data, we constructed a diagnostic model for MDD patient with 16 indicators, achieving an AUC of 0.973 in the internal validation set. Additionally, a treatment response prediction model based 7 cytokines was developed, resulting in an AUC of 0.944 in the internal validation set. Furthermore, Mfuzz time-series analysis revealed that cytokines such as Basic fibroblast growth factor (bFGF), Interleukin 13 (IL-13), and Interleukin 1 receptor, type I (IL1R1) that revert towards normal levels after 8 weeks of treatment, suggesting their potential as therapeutic targets for MDD.

**Conclusions:** Our diagnostic model derived from CyTOF and cytokines demonstrates high diagnostic value. However, relying solely on immune cells may not provide optimal predictions for antidepressant treatment response. In contrast, leveraging cytokines has proven valuable, leading to the construction of a seven-factor treatment response prediction model. Importantly, we observed that several significantly altered cytokines in MDD can normalize following antidepressant treatment, indicating their potential as therapeutic targets.

## Introduction

Major depressive disorder (MDD) is a pervasive public health concern, affecting over 300 million people across the globe. The World Health Organization anticipates that by 2030, MDD may become the primary source of disability worldwide 1. However, despite its considerable impact, the precise pathophysiological mechanisms of this severe ailment are yet to be fully comprehended. Presently, the diagnosis of MDD and the choice of medication depend heavily on the physician's clinical experience and evaluations using specific scales 2. The lack of stable and reliable objective biomarkers presents an obstacle for healthcare professionals when it comes to precise diagnosis and determining efficient treatment strategies. Consequently, there is an urgent need for objective biomarkers that can not only assist in diagnosing but also contribute to personalized treatment approaches for patients, thereby eliminating trial-and-error in drug administration.

In recent years, a wealth of research has suggested that the inflammation may be related to the pathogenesis and antidepressant treatment of MDD 3-5. Certain cytokines can participate in cellular signal transduction, immune response, and reactions to internal and external factors 6. These small cytokines include interleukins, interferons, chemokines and tumor necrosis factors 6. Numerous studies have reported increased levels of peripheral blood cytokines, including IL-6, IL-10, and TNF-α, in patients with MDD 7,8. Some findings also suggested these inflammatory factors, TNF-α, IL-6, IL-12 and IL-4 have potential as objective biomarkers for diagnosing MDD 9,10. In addition to cytokines, significant changes in immune cells are also found in MDD. This includes increased B cells, natural killer cells, Th1/Th2 ratio and significantly reduced Treg cells 11-13.

Our previous study found noteworthy differences in immune cell profiles between MDD patients and healthy controls (HC), including increased proportions of CCR2+ CD8T cells, Trem1+ monocytes, IgD+ memory B cells, and plasmablasts, as well as decreased levels of Tregs, follicular T cells, gamma-delta T cells, Th17 cells, naïve CD4 T cells, and CD4 T cells 14. Additionally, immune cells and cytokines associated with the MDD pathogenesis may relate to antidepressant treatment response 8,15. For example, a decrease in the pro-inflammatory cytokines IL-1β concentration and an increase in Treg cells were observed after treatment 16. Some cytokines, such as IL-8, have been suggested as predictors of antidepressant effectiveness, while elevated plasma levels of IL-1β and BDNF have been identified as potential predictors for resistant depression patients 17-19. Furthermore, certain immune cells like circulating cytotoxic T cells and natural killer cells may act as predictors for antidepressant response in melancholic depression 20. However, limitations such as small sample size, the low sensitivity in detection method, restricted range of analyzed cytokines and immune cell subsets and lack of longitudinal research have constrained the reproducibility and sensitivity of these investigations.

A deeper understanding of the diverse functions of immune cell subsets could shed light on treatment response in MDD. Traditional fluorescent flow cytometry falls short due to its inability to simultaneously analyze multitude features. Mass cytometry by time-of-flight (CyTOF), capable of measuring up to 50 features in a single cell, has been extensively used in cancer and other neurologic disease research such as solid tumors, Alzheimer's and Parkinson's diseases, to overcome this limitation 21-23. Moreover, currently, CyTOF is being employed to investigate the relationship between various cell subpopulations and clinical responses, such as predicting the efficacy of immunotherapy in multiple types of cancer 24,25. However, the use of CyTOF in MDD research remains limited. While we have previously utilized CyTOF to discern differences in the peripheral blood immune cell spectrum between patients with MDD and HC 14, there is still much unexplored potential for systematic exploration using CyTOF to elucidate diagnostic and therapeutic strategies for MDD. Single or few indicators may not be sufficient for diagnosing or predicting treatment outcomes in complex diseases like MDD. The combination of multi-dimensional omics data holds promise for better diagnostic and prognostic value. Despite current high-throughput detection methods can measure hundreds of cytokines and immune cells in clinical samples, data processing and interpretation remain challenging.

Machine learning, a widely adopted artificial intelligence technique, is extensively employed for automated analysis of complex data across various biomedical domains, offering distinct benefits in interpreting omics data, identifying biomarkers, and constructing predictive models for precision medicine 26,27. However, there remains a substantial gap in applying machine learning to analyze cytokine and immune cell data in MDD for developing diagnostic and therapeutic prediction models. Therefore, this study aims to systematically detect cytokines and immune cells through high-throughput methods, integrate these data types, and develop diagnostic and therapeutic prediction models using various machine learning techniques to evaluate their value in the diagnosis and therapeutic prediction for MDD. Furthermore, we explored the dynamic changes in cytokines in MDD patients before and after treatment, identifying potential factors that could serve as therapeutic targets.

## Materials and Methods

### Participants and procedure

The iMore study is a prospective observational cohort study entitled “Integrated Module of Multidimensional Omics for Peripheral Biomarkers (iMORE) in Patients with Major Depressive Disorder 28.” This study has been approved by the research ethics committees at the Shanghai Mental Health Center in China. The ethical approval number is 2020-87. Prior to the study, written informed consent was obtained from all participants. Furthermore, the study was registered in the Clinical Trials registry under the identifier NCT04518592. The samples utilized in this study represent a subset of those collected for the iMore study, all of which were obtained from the Shanghai Mental Health Center. Moreover, the patients and HC included in this analysis were recruited from December 2020 to June 2022. Patients or the public were not involved in the design, or conduct, or reporting, or dissemination plans of our research. This study incorporated 134 patients with MDD and 50 HC. Patients were evaluated and had peripheral blood samples collected at baseline, Week 4 (W4), and Week 8 (W8), while the HC underwent evaluation and blood sampling only at baseline. All of the 50 HC completed both baseline CyTOF detection and cytokine testing. Among the 134 patients who underwent the baseline CyTOF detection, 84 also completed the baseline cytokine testing. However, one patient's CyTOF test and cytokine test results were excluded due to very low detection. Therefore, a total of 133 baseline CyTOF results and 83 baseline cytokine results were available for diagnostic model modeling analysis.

At W4, 90 patients completed the CyTOF detection and 59 finished cytokine testing. By W8, 79 patients completed CyTOF detection and 35 finished cytokine testing. This study included adult patients between the ages of 18 and 65 who met the diagnostic criteria for MDD according to the Diagnostic and Statistical Manual of Mental Disorders, 5th Edition. The eligibility criteria were as follows: (1) adults primarily diagnosed with MDD, either experiencing their first episode or a recurring one; (2) a baseline Montgomery-Asberg Depression Rating Scale (MADRS) total score of ≥24; (3) a baseline HAMD-17 total score of ≥20; (4) voluntary provision of informed consent. The exclusion criteria were primarily: (1) current presence of any Axis I disorder apart from MDD; (2) serious suicide risk, or a score greater than 3 on suicidal thoughts (MADRS item 10); (3) severe physical illnesses like cerebrovascular disease, respiratory disease, blood disorders, liver and kidney diseases, endocrine disorders, nervous system disorders, among other systemic diseases; (4) pregnancy or lactating phase. More comprehensive exclusion details can be referenced in the previously published article 28.

### Mass cytometry analysis of blood immune cells and data analysis

Firstly, peripheral blood mononuclear cells (PBMCs) were segregated from human blood samples that had been collected in K2-EDTA tubes. Each cell sample was then distinctly labelled with a barcode isotope for 30 minutes. Post-labeling, the PBMCs were washed, resuspended in deionized water, and amalgamated with 20% EQ beads. Staining of cells was carried out using metal-labelled antibodies, following the guidelines provided by the manufacturer (Fluidigm Science, lnc). Cells were subjected to viability staining by treating them with Cisplatin at a final concentration of 0.5 µM. Prior to surface staining, Fc receptors were blocked by incubating the cells with a blocking antibody for 10 minutes at room temperature. A surface antibody cocktail was subsequently added and left to stain for 30 minutes on ice. Prior to intracellular staining, the cells were rinsed with a staining buffer and then fixed with a 1.6% paraformaldehyde solution at room temperature for 10 minutes. This was followed by a rinse with perm-S. The intracellular antibody cocktail was then introduced and allowed to stain for another 30 minutes on ice. After a final wash with perm-S, the cells were resuspended in 1 ml of Ir-Interchelator within Fix/Perm buffer and incubated overnight at 2-8 °C. Prior to acquisition, the cells were prepared in a Cell Acquisition Solution containing EQ Four Element Calibration beads diluted to 1:10 and filtered through a 35 µm nylon mesh filter cap. Finally, cell acquisition was performed on a Helios Mass Cytometer at an event rate of 200-300 events per second with a total count of around 3x10^5^ cells. The data files were subsequently exported and examined using the analytical tools provided by Cytobank software (https://www.cytobank.org/). The frequency of annotated cell populations was evaluated using a t-test or Mann-Whitney test, depending on the normality of the distribution. Details of the applied antibodies can be referred to in our previously published article 14.

### Quantibody array and data analysis

The assessment of 440 cytokines was carried out utilizing a sandwich approach hinged upon the antibody array technique (Human Cytokine Antibody Array, RayBiotech, Norcross, GA, USA). In essence, 100μL of plasma, following a 1:2 dilution, was introduced to a chip embedded with 440 primary antibodies and allowed to incubate at 4℃ overnight. After comprehensive washing steps, biotin-labelled antibodies were added for a duration of 2 hours, succeeded by another round of washing. After this, an 80 µL aliquot of Cy3-conjugated streptavidin was dispensed, followed by a 1-hour incubation period in darkness at ambient temperature. Following an additional wash cycle, the fluorescence signal data specific for Cy3 was captured via the InnoScan 300 Microarray scanner (Innopsys). These acquired data points were later converted into quantitative values utilizing Mapix software tools. The data were normalized against positive controls. The data from the human cytokine antibody array were analyzed using either the independent t-test or the Mann-Whitney U test, based on whether the distribution was normal or not. P < 0.05, log_2_FC = 0.25 was considered statistically significant.

### Diagnostic model construction

Among the MDD patients, a total of 84 individuals had complete plasma and cytokine data; however, one patient's CyTOF test and cytokine test results were excluded due to very low detection, resulting in 83 individuals being included in the construction of the diagnostic model. Initially, the immune cells and cytokines were selected via three machine learning algorithms: logistic regression, least absolute shrinkage and selection operator (LASSO) regression employing a 10-fold cross-validation method, and recursive feature elimination (RFE) with a random forest classifier, also using a 10-fold cross-validation approach. Moreover, patients and controls with both CyTOF and cytokine detection data were randomly assigned into training and testing sets at a 7:3 ratio. The overlapping cytokines and immune cells, as jointly discerned by the three algorithms, served as the foundation for constructing a predictive model for MDD within the training set. This model was built utilizing eight classical machine learning algorithms: an elastic network (Enet), ridge regression, LASSO, gradient boosting machine (GBM), random forest (RF), supervised principal components (SuperPC), eXtreme Gradient Boosting (XGboost), and a support vector machine (SVM). Subsequently, models were evaluated using indices such as area under the curve (AUC), accuracy rate, recall and F1-score based on both the training and testing datasets. Further validation of the model's performance was conducted in the test set, which led to the selection of the optimal model (MDD risk score = Expression of CXCL14 × 0.93 + Expression of VEGFR1 × 0.47 + Expression of IL-1 RII × 0.15 + Expression of RANK × 0.09 + Expression of XEDAR × -0.20 + Expression of GITR × -0.36 + Expression of CRTAM × -0.47 + Expression of IL-11 × -1.15 + Expression of IGFBP-2 × -1.15 + Expression of Fetuin A × -1.16 + Proportion of Trem1 classical Monocytes × 1.16 + Proportion of B cells × 0.18 + Proportion of CD36 Monocytes × 0.07 + Proportion of HLADR Monocytes × -0.06 + Proportion of Th17 × -0.61 + Proportion of Treg × -1.60). Shapley Additive Explanations (SHAP) analysis was used to interpret the model. SHAP analysis was performed using the iml package in R. The Hosmer-Lemeshow test was used to assess the goodness of fit of this model.

### Treatment response prediction model construction

Patients were stratified into response and non-response groups based on the 8-week treatment outcome, specifically whether the reduction rate on the MADRS scale was equal to or greater than 50%. Given that no significant differences in immune cells at baseline were detected between these two groups, cytokine data was primarily used for subsequent predictive model construction. Initially, univariate logistic regression was applied to identify cytokines correlated with antidepressant treatment response, resulting in seven cytokines. Following this, eight classical machine learning algorithms were executed to construct a MDD treatment efficacy prediction model. These included an Enet, LASSO, ridge regression, GBM, RF, SuperPC, XGboost, and SVM. Given the limited number of samples for both response and non-response groups, bootstrap resampling was performed 100 times on all of the samples to create the test set. This allowed for the calculation of accuracy, AUC, recall and F1-score for each model, leading to the identification of the most optimal response prediction model (Response score = Expression of CD163 × 0.74 + Expression of BMPR-IA × 0.56 + Expression of Leptin R × 0.33 + Expression of OPN × 0.33 + Expression of PF4 × 0.10 + Expression of ACE-2 × 0.10 + Expression of Syndecan-1 × 0.04). It's worth noting that the model constructed based on XGboost displayed evident overfitting and was therefore excluded from subsequent analyses.

### Mfuzz analysis

Initially, using the one-way analysis of variance (ANOVA), cytokines significantly differentially expressed across four groups (comprising 'HC', 'pre-treatment for MDD', '4 weeks post-treatment for MDD', and '8 weeks post-treatment for MDD') were identified. Following this, the Mfuzz package was utilized for clustering analysis on the expression patterns of these identified cytokines according to their sequential order through the stages. The cytokines exhibiting synchronous changes in expression patterns throughout all stages, which reflect disease progression and treatment process, were then identified. These factors, inferred to be closely associated with the response to antidepressant treatment, were incorporated into subsequent analyses aimed at constructing a treatment response prediction model.

### Statistical analysis

Depending on the normality of continuous variables, comparisons between two groups were made using either independent samples t-test or Mann-Whitney U test (two-tailed). For multiple group comparisons, ANOVA was applied based on the normality of distribution. Categorical variables were compared using Chi-square test. R software (v.4.1.0) was used for analysis and graphical representation. P-value < 0.05 was considered statistically significant with two sides. Odds ratios (ORs) and 95% confidence intervals (CIs) were reported if necessary.

## Results

### Comparison of immune cell characteristics between patients with MDD and HC

**Figure 1:**
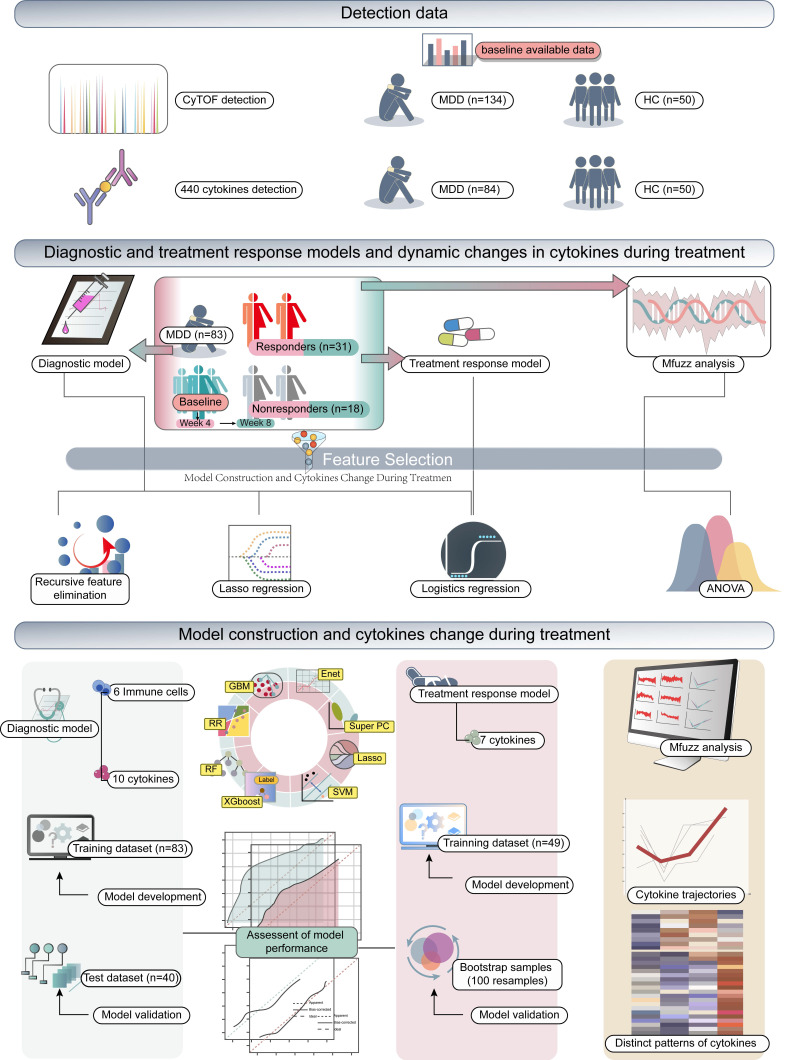
Schematic overview of the study.

**Figure 2:**
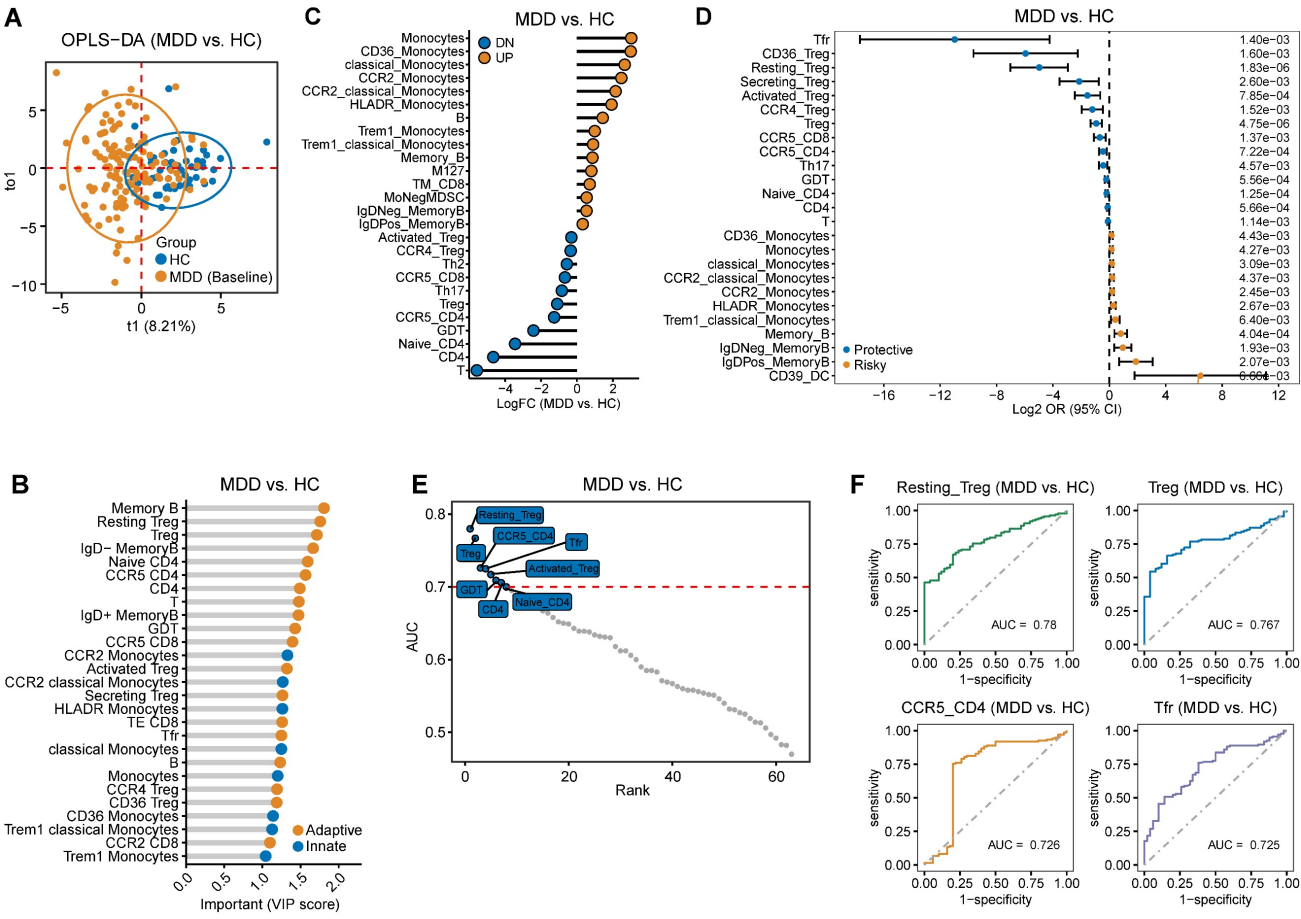
** Difference in immune cell characteristics between patients with Major depressive disorder (MDD) and HC.** (A) Orthogonal partial least squares discriminant analysis (OPLS-DA) based on immune cell characteristics shows separation between MDD and HC groups. (B) The dots representing immune cells are selected based on the VIP score (>1, OPLS-DA). The yellow dots represent adaptive immune cells, while the blue dots represent innate immune cells. (C) Significant differences in immune cells between patients with MDD and controls. (D) Identification of top 25 immune cells associated with MDD using univariate logistic regression analysis. (E) Immune cells with an Area Under the Curve (AUC) ≥ 0.7, indicating their diagnostic value. (F) Top 4 immune cells based on AUC ranking.

The overall workflow of this study was illustrated in (Fig. 1). We performed a comparison of immune cell subsets in PBMCs between individuals with MDD and HC. Demographic data and clinical characteristics are presented in Table S1. Using the Orthogonal Partial Least Squares Discriminant Analysis (OPLS-DA) models, we observed a clear separation between the two groups (R^2^ = 0.4, Q^2^ = 0.24) as depicted in Figure 2A. Immune cell subsets with a Variable Importance in Projection (VIP) score ≥ 1 were considered discriminative, and these subsets are highlighted in Figure 2B. In addition, significant differences in immune cells between patients with MDD and controls were revealed (Figure 2C). These results indicate that in patients with MDD, CD4+T cells including Treg, Th2, and Th17 cells are significantly reduced, while monocytes and B cells are significantly increased. Further, we employed logistic regression analysis to examine whether immune cells are risk factors for MDD. The results showed that monocytes and B cells are risk factors for MDD, while Tfr, Treg cells, and Th17 cells are protective factors against MDD (Figure 2D). Furthermore, we used ROC curve analysis to evaluate the diagnostic value of immune cells, and we displayed immune cells with an AUC greater than or equal to 0.7 (Figure 2E), with the top 4 cells being Resting T, Treg cell, CCR5+ CD4+ T, and Tfr cells (Figure 2F). This suggests that immune cells may have potential diagnostic value.

### Comparison of cytokines between patients with MDD and HC

**Figure 3:**
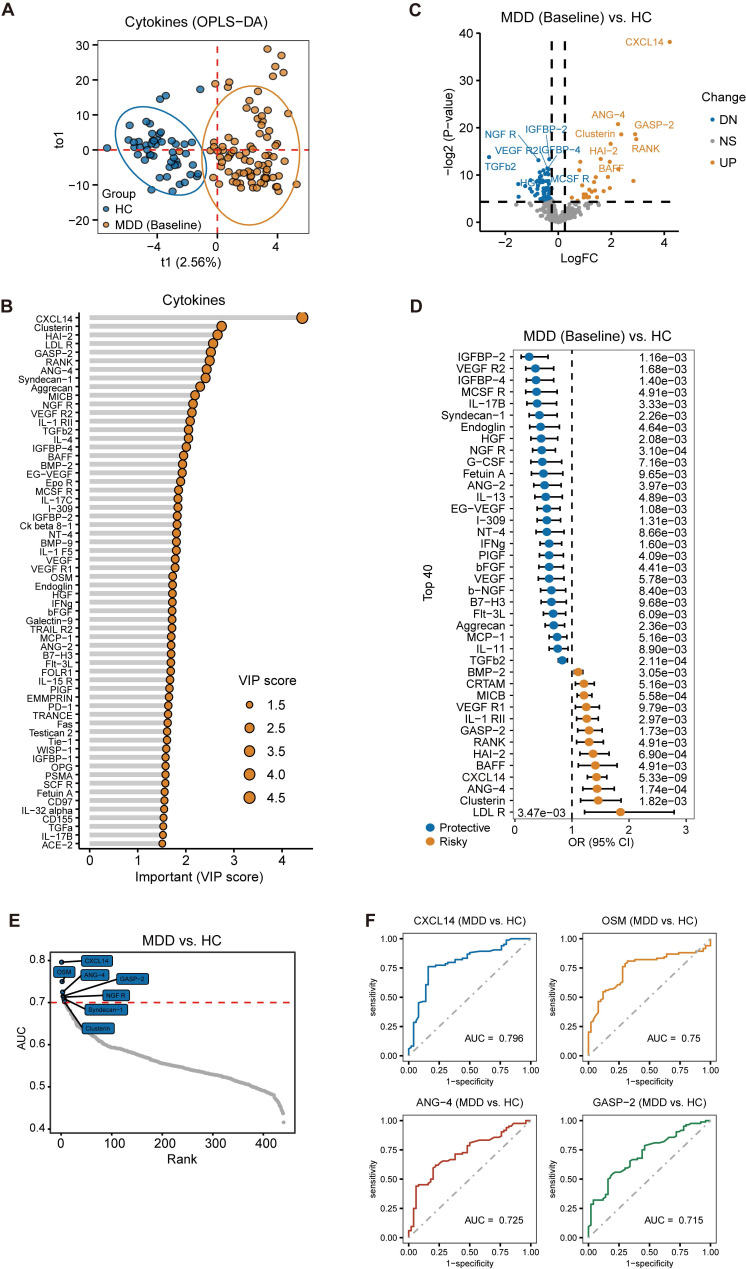
** Cytokine differences between patients with Major Depressive Disorder (MDD) and HC.** (A) Clear distinction between MDD and HC groups based on cytokine using OPLS-DA model. (B) Cytokine selection based on VIP score (>1, OPLS-DA). (C) Significant cytokine level differences found between MDD patients and controls. (D) Risky or protective cytokine identification for MDD using univariate logistic regression model analysis. (E) Cytokines exhibiting a diagnostic value indicated by an Area Under the Curve (AUC) ≥ 0.7 (F) Top 4 Cytokines ranked by AUC.

Table S2 presents the demographic data and clinical characteristics of the study cohort, which includes individuals who underwent cytokine detection. The OPLS-DA model demonstrated a clear distinction between the MDD and HC groups, as evidenced by an R^2^ value of 0.801 and a Q^2^ value of 473 (Figure 3A). Furthermore, the analysis identified cytokines with a VIP score greater than 1, highlighting their potential significance (Figure 3B). Additionally, significant differences in the levels of some cytokines were found between MDD patients and controls (Figure 3C). Moreover, logistic regression analysis was applied to explore the relationship between cytokines and MDD. The results showed that certain nutritional factors, such as Insulin-Like Growth Factor Binding Protein 2 (IGFBP-2), emerged as the significantly protective factors for MDD. Additionally, several neurotrophic factors previously reported to be closely associated with MDD, such as bFGF, vascular endothelial growth factor (VEGF) and nerve growth factor (NGF), are also considered protective factors for MDD. Conversely, the low density lipoprotein receptor (LDLR), Clusterin, Angiogenin-4 (ANG-4), and C-X-C motif chemokine ligand 14 (CXCL14) were risk factors for MDD (Figure 3D). Furthermore, ROC curve analysis was performed to assess the diagnostic value of cytokines. The results presented cytokines with an AUC greater than or equal to 0.7 (Figure 3E), with the top 4 factors being CXCL14, Oncostatin M, ANG-4, and Growth and differentiation factor-associated serum protein-2 (GASP-2) (Figure 3F). These findings also indicate the potential diagnostic value of cytokines for MDD.

### Constructing and evaluating a diagnostic model based on the combination of immune cells and cytokines

**Figure 4:**
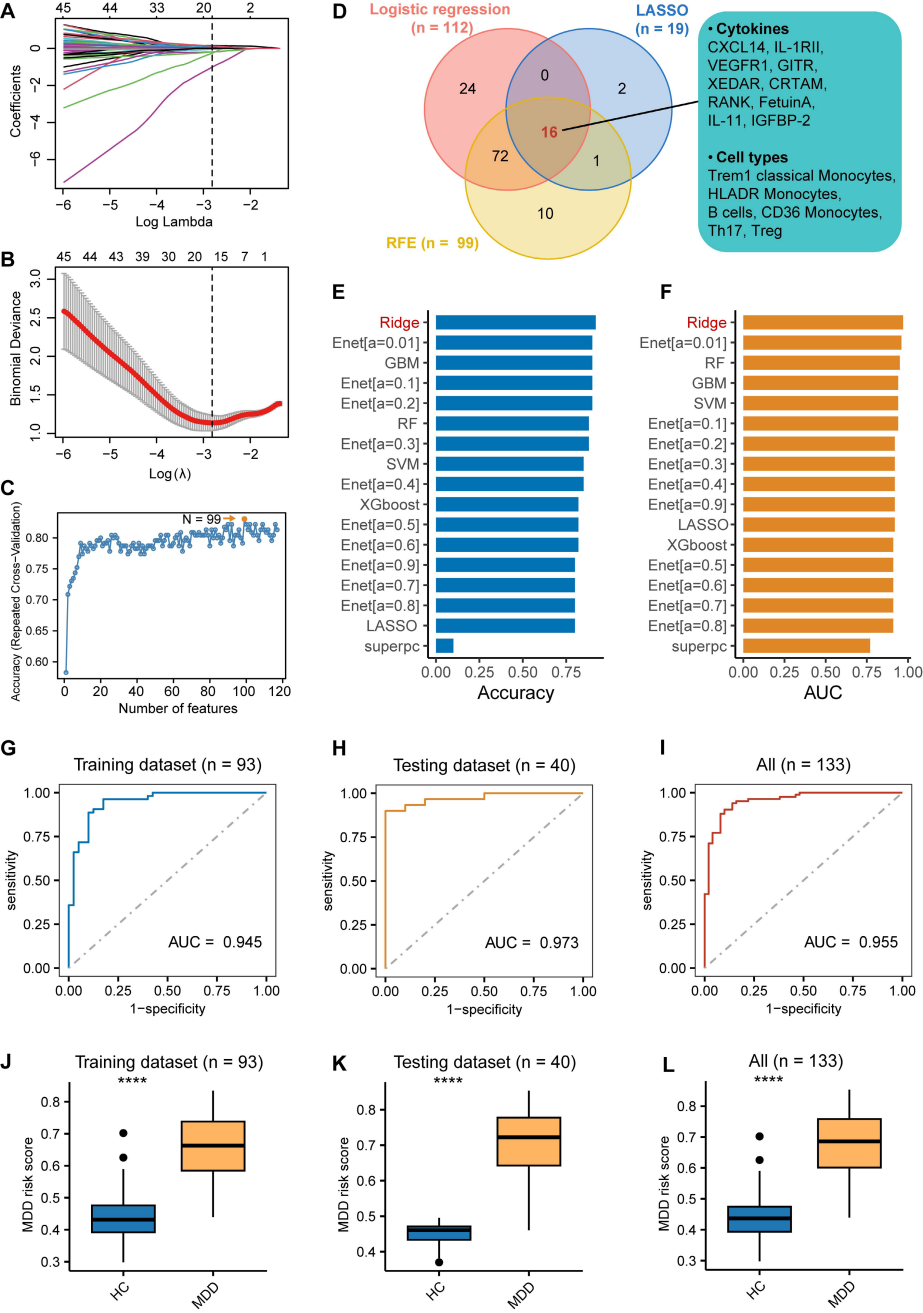
** Diagnostic model construction and evaluation based on immune cell and cytokine combination.** (A-B) LASSO regression with the lambda.min as the cutoff threshold. (C) The feature selection method of recursive feature elimination (RFE). (D) Overlap of significant features identified by univariate logistic regression, LASSO regression, and RFE algorithm visualized using a Venn diagram. (E-F) High accuracy rates and Area Under the Curve (AUC) values achieved by eight algorithms, with ridge regression analysis exhibiting the highest accuracy rate and AUC value simultaneously. (G-I) AUC by Ridge Regression Algorithm in Training Data, Test Data, and All Data. (J-L) Significant differences in MDD risk scores between MDD patients and the healthy control group observed in the training dataset, testing dataset, and across all participants.

In previous research, it has been suggested that diagnostic efficacy may be relatively low when using a single indicator or a single omics indicator as a diagnostic biomarker. Thus, our aim was to explore the potential of generating diagnostic biomarkers by combining cell type abundance detected by CyTOF and cytokine expression profiles. To this end, we designed a pipeline to construct a diagnosis model for MDD using multiple machine learning algorithms. Initially, previous univariate logistic regression model has been used to identify risky or protective immune cell and cytokine levels for MDD, resulting in 31 out of 63 significant cell types and 81 out of 440 significant cytokines (P < 0.05, Table S3). Furthermore, differential expression analysis identified 26 differential cell types and 97 differential cytokines between MDD and HC (P < 0.05, Table S4). The LASSO regression with lambda.min as cutoff (Figure 4A-B) and Recursive Feature Elimination (RFE) algorithm with a random forest classifier (Figure 4C) were then utilized on these differentially expressed features to pinpoint the key diagnostic features. A Venn diagram was assembled to visualize the overlap of the significant features identified by the univariate logistic regression, LASSO regression, and RFE algorithm, revealing a total of 16 overlapping features as the most critical diagnosis features, which included 6 cell types and 10 cytokines (Figure 4D). This dataset was randomly divided into a training set consisting of 58 MDD patients and 35 controls, and a test set comprising 25 MDD patients and 15 controls. The division ratio between the training and test sets was maintained at 7:3. Based on the 16 most important diagnostic features, eight classical machine learning algorithms - Enet, ridge regression, LASSO, GBM, RF, SuperPC, XGBoost, and SVM - were employed to construct MDD diagnostic models using the training set. Lastly, the best model was identified by evaluating indices such as area under the curve (AUC), accuracy rate, recall and F1-score based on the test set.

The results demonstrated that all algorithms achieved high accuracy rates and AUC values, with ridge regression analysis simultaneously displaying the highest accuracy rate and AUC value (Figure 4E-H). In the training set, the AUC of the ridge regression was 0.945, accuracy was 0.900, recall was 0.946, and F1-score was 0.959. In the test dataset, the AUC of the ridge regression was 0.973, accuracy was 0.920, recall was 0.846, and F1-score was 0.759. The recall and F1-score of all models are presented in Figures S1A and S1B. Moreover, this high level of discrimination was also observed among all patients (Figure 4I). The SHAP values for each variable in the diagnostic model were calculated to assess their feature importance and are presented (Figure S1C). The Hosmer-Lemeshow test, with a P-value of 0.74, indicated a good calibration of our diagnostic model in the test set (Figure S1D). Additionally, an MDD risk score was obtained for each patient by using the model and calculating based on each patient's CyTOF and cytokine values. Significant differences in these MDD risk scores between patients and the healthy control group were observed in the training dataset, testing dataset, and across all participants (Figure 4J-L). In summary, these results suggest that this diagnostic model exhibits high performance.

### Analysis of baseline immune cell characteristics in responders and non-responders

**Figure 5:**
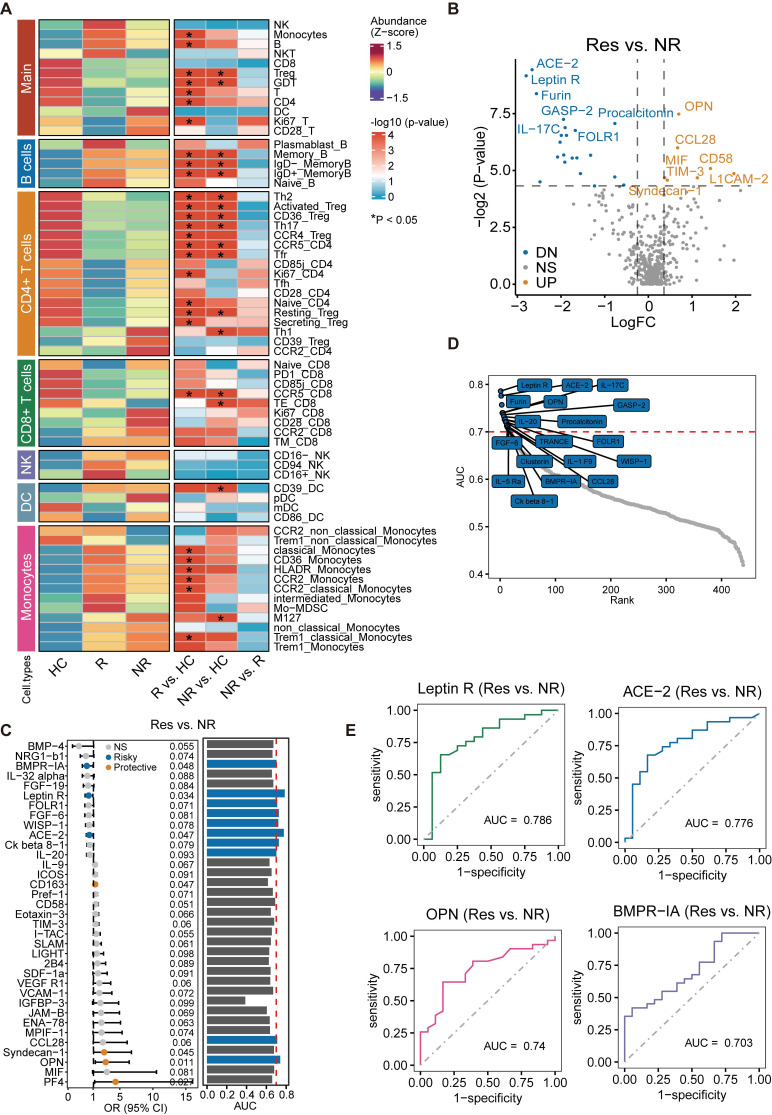
** Comparative analysis of baseline immune cell characteristics and cytokine levels between responders and non-responders.** (A) No significant differences observed in baseline immune cell levels between responders and non-responders. (B) Volcano plot illustrating significant difference in cytokines in responders compared to non-responders. (C) Exploration of the relationship between cytokine levels and treatment response. (D) Area Under the Curve (AUC) >0.7 for seven treatment response-associated factors. (E) Predictive value assessment of the top four cytokines ranked by AUC for treatment response.

Although significant differences in baseline immune cells were found between both the responders and HC, and the non-responders and controls, no significant differences were observed when comparing the baseline levels of immune cells between responders and non-responders (Figure 5A). The relevant clinical information of responders (n = 56) and non-responders (n = 28) has been included in Table S5. This suggests that the predictive value of baseline immune cell levels for the efficacy of antidepressant treatment may be limited. Therefore, we further investigated whether baseline cytokine levels could serve as a predictor for the effectiveness of antidepressant drug treatments.

### Differences in baseline cytokine levels between responders and non-responders

Based on the distribution of the data, we utilized a t-test or Mann-Whitney U Test with P < 0.05 and Log_2_FC = 0.25 as threshold criteria. Table S6 contains the relevant clinical information of individuals who completed cytokine detection, categorized as treatment responders (n = 31) and non-responders (n = 18). The results were represented in a volcano plot, where we observed significant elevations in cytokines such as OPN, CCL28, CD58, MIF, and TIM-3 in treatment responders. In contrast, levels of Angiotensin-converting enzyme 2 (ACE-2), Leptin Receptor (leptin R), IL-17C, and GASP-2 were significantly decreased (Figure 5B). A considerable number of these factors are intimately linked with immune-inflammatory responses or their regulation. Moreover, further exploration of the relationship between cytokine levels and treatment response through logistic regression analysis revealed that increased levels of OPN, PF4, CD163, and Syndecan-1 can predict a positive response to treatment. Conversely, elevated levels of ACE-2, leptin R, and BMPR-1A indicate a lack of response (Figure 5C). The ROC curve analysis presented seven factors associated with treatment response, each with an AUC ranking greater than 0.7 (Figure 5D). Further demonstration was performed on the predictive value of the top four factors ranked by AUC. Remarkably, leptin R, when considered as a single factor, displayed the highest predictive value for treatment efficacy, with an AUC of 0.786 (Figure 5E).

### Analysis of combined predictive value of cytokines for treatment response

**Figure 6:**
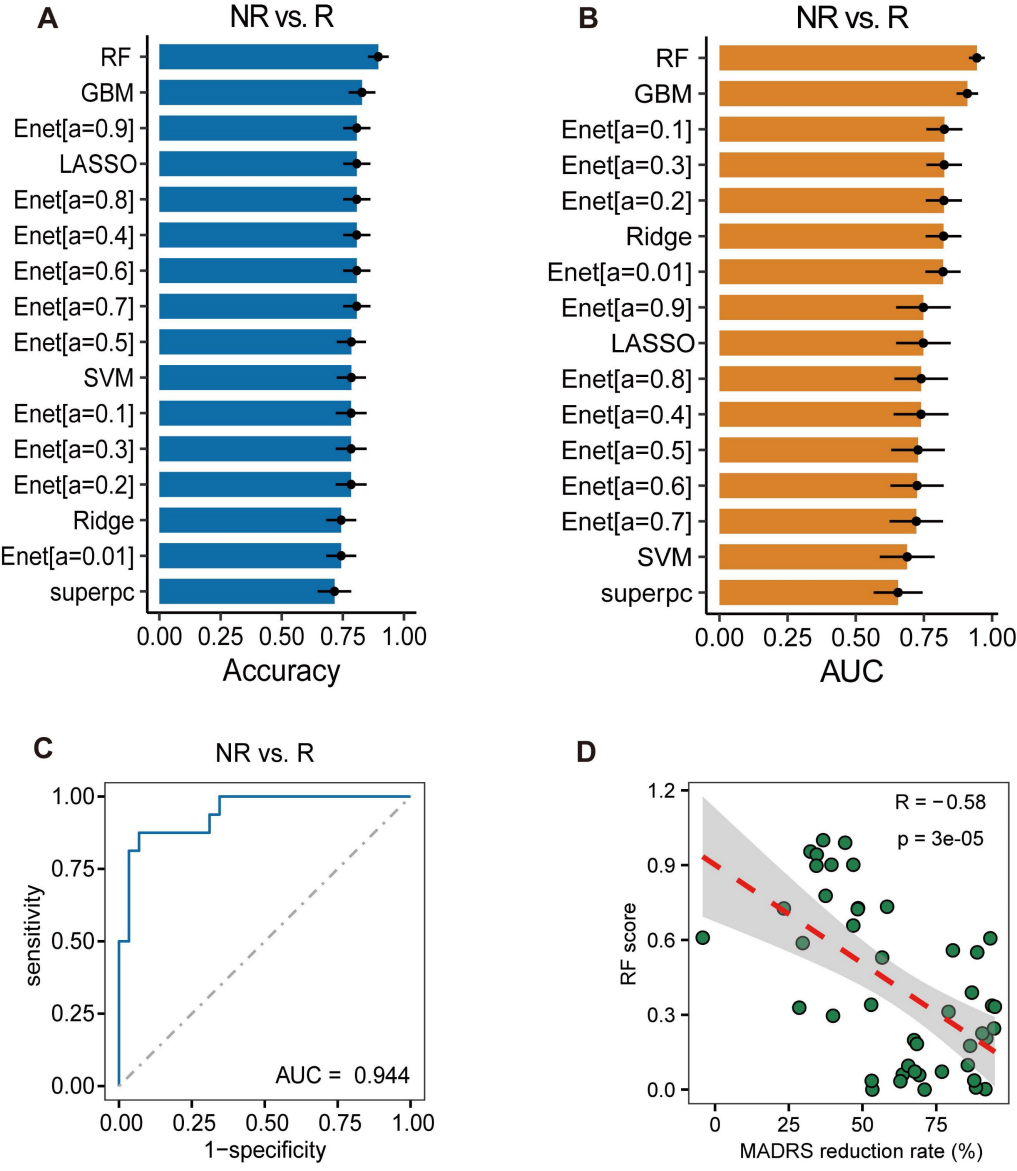
** Analysis of Combined Predictive Value of Cytokines for Treatment Response.** (A) Accuracy of different machine learning models tested via bootstrap resampling. (B) Area Under the Curve (AUC) of different machine learning models tested via bootstrap resampling. (C) Random Forests (RF) model outperforms other algorithms with highest Average AUC score. (D) Correlation between response score computed using RF model and percentage reduction in MADRS score.

The results mentioned above highlight the limited capability of baseline CyTOF results in predicting antidepressant response. Furthermore, when predicting on cytokine factors, the highest diagnostic value observed in single-factor analysis did not exceed 0.8. Consequently, we attempted to construct a treatment response prediction model by combining multiple cytokine factors. Employing the seven factors previously identified as relevant to treatment response, different machine learning methods were utilized for model construction. Each model was tested via bootstrap re-sampling performed 100 times, and the accuracy (Figure 6A) and AUC (Figure 6B) for each model were calculated. The RF model showed the highest average accuracy of 0.895 ± 0.004 and an AUC of 0.944 ± 0.003 (Figure 6C), along with a recall of 0.938 ± 0.004 (Figure S2A) and an F1-score of 0.919 ± 0.003 (Figure S2B), thereby demonstrating good predictive value. The SHAP values for each variable in the treatment response prediction model are presented (Figure S2C). The Hosmer-Lemeshow test showed a P-value of 0.88, indicating good calibration of treatment response prediction model (Figure S2D). Additionally, using the RF model for treatment response prediction, a response score was computed for each patient, revealing a significant correlation between this score and the percentage reduction in the MADRS score (Figure 6D). These findings suggest that combining multiple cytokine factors offers superior predictive value compared to single-factor leptin R, which has the highest AUC.

### Dynamic changes in cell abundance and cytokine expression during drug treatment

**Figure 7:**
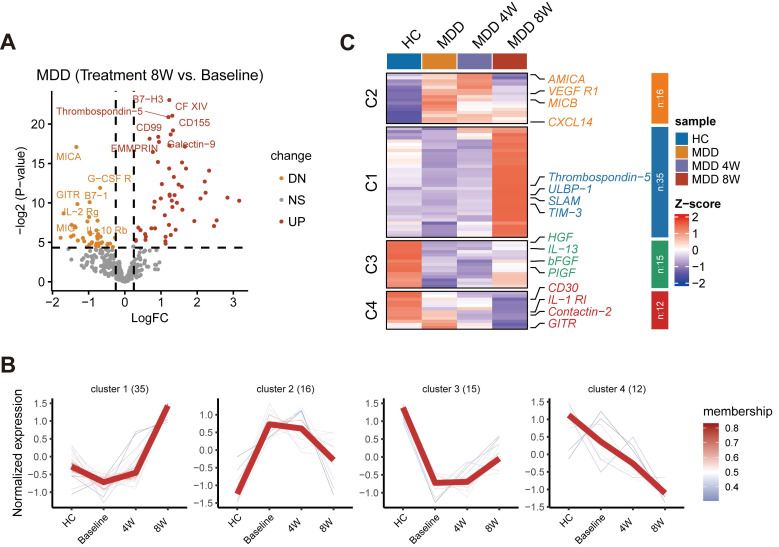
** Investigation of changes in cytokine expression during anti-depressant treatment.** (A) Volcano plot illustrating changes in cytokine levels before and after 8 weeks of treatment. (B) Cluster analysis by the Mfuzz method identifying four main clusters of longitudinal trajectories during the treatment course. (C) Subset of significant cytokines with distinct clusters.

Given our observation that baseline cytokine levels might be associated with the efficacy of anti-depressant treatment, we further investigated the dynamic changes in cytokine expression during drug therapy. A volcano plot illustrated the changes in cytokine levels before and after 8 weeks of treatment (Figure 7A). Cytokines that significantly changed during the 8-week treatment were further examined using cluster analysis by the Mfuzz method. Four main clusters of longitudinal trajectories were identified, delineating different patterns during the treatment course (Figure 7B). A subset of significant cytokines was identified showing distinct patterns, including VEGFR1, TIM-3, bFGF, CD30, and other factors (P < 0.05) (Figure 7C). Many of these factors are neurotrophic or immune-inflammatory related, and some have been previously reported to be associated with MDD. Importantly, following treatment, these cytokines, especially in Custers 1-3, tended to normalize to levels observed in HC. Therefore, these factors may represent potential targets for the therapeutic action of anti-depressant treatment.

## Discussion

To date, our study is the first to systematically explore the potential of immune cells and cytokines as diagnostic and therapeutic efficacy predictive markers, using a combined approach of CyTOF and cytokine analysis. In this study, we applied CyTOF technology to compare the differences among 63 peripheral immune cells between patients with MDD and controls. The OPLA analysis suggested that immune cells could significantly distinguish between patients and HC. Among these 63 cell types, significant differences were found in 26 kinds of immune cells. In particular, significantly elevated levels of peripheral blood monocytes and B cells were observed in patients with MDD, whereas Treg, Th2, Th17, and other cell populations demonstrated notable reductions. These results are similar to those reported in our previous study 14, and similar conclusions have been drawn by some studies using flow cytometry 12,29,30.

It is noteworthy that this study is the largest CyTOF testing study for peripheral samples in the field of MDD research to date, and it is also the first to use peripheral immune cell data to explore their potential as diagnostic and therapeutic markers for MDD. CyTOF enables the simultaneous detection of a larger number of immune cells compared to flow cytometry. This expanded capability allows for comprehensive screening of peripheral immune cells and facilitates the development of diagnostic and predictive models for antidepressant efficacy based on immune cell profile data. The AUC indicators for the top four ranked immune cell types, including Resting T, Treg, CCR5+ CD4+ T, and Tfr cells, were all greater than 0.7, suggesting that these cell types have a certain diagnostic value for MDD. Furthermore, we analyzed the differences in peripheral blood cytokines between patients with MDD and HC and further explored the potential of cytokines as diagnostic markers. Our findings, which showed lower VEGF, bFGF, Fetuin A, and HGF in patients with MDD, were consistent with those reported in previous studies 31-34. Additionally, this study also made new discoveries. For example, we reported that plasma IGASP-2, CXCL14, OSM, ANG-4 showed significant differences in MDD and ROC analysis demonstrated that they have a moderate diagnostic value. Previous studies have not found that these plasma protein levels are related to MDD. Importantly, in this study, we used several machine learning algorithms and combined CyTOF and cytokine data to build a diagnostic model containing 16 indicators, including six types of immune cells and ten cytokines. The AUC of this model on both the training set and test data was over 0.9, indicating it has high diagnostic significance.

Previous studies have found that immune profiling can predict treatment response in some diseases such as multiple sclerosis and Melanoma and using CyTOF technology 35,36. Thus, we further tried to construct a therapeutic prediction model. However, in this study, we did not find that baseline immune cells could predict the efficacy of 8-week antidepressant treatment. To our knowledge, only one study has used flow cytometry analysis to detect and further explore the role of baseline immune cells in predicting antidepressant treatment. The results showed that NK cells and circulating cytotoxic T cells could predict the efficacy of antidepressants in patients with melancholic depression 20. These findings suggest that different subtypes of MDD may have specific biomarkers associated with treatment response. Unfortunately, since we did not assess MDD subtypes in our study, we were unable to validate these subtype-specific markers.

It should be pointed out that there is still very little research on the use of peripheral blood immune cells to predict the efficacy of antidepressant treatment, and our research provides new data. Our findings suggest that immune cells may not serve as ideal biomarkers for predicting therapeutic efficacy. Therefore, we further utilized cytokines to explore their potential predictive value for antidepressant efficacy. We constructed a seven-factor efficacy prediction model and found that it had high predictive value. Levels of plasma OPN, PF4, CD163, Syndecan-1, ACE-2, leptin R, and BMPR-1A have not been previously reported to predict the therapeutic effects of antidepressants. Interestingly, a clinical trial targeting patients with MDD showed that the G8790A genetic variant of ACE2 correlated better with the efficacy response to SSRIs 37. ACE2 can degrade ANGII to generate angiotensin, and it has been reported that the action of AngII is reduced by antidepressants 38. Therefore, it cannot be ruled out that ACE2 gene polymorphism might affect the treatment outcome of antidepressants by influencing peripheral blood plasma ACE2 levels. These results further support that the renin-angiotensin system may be closely related to the action of antidepressants. Leptin R has been considered a target for antidepressant treatment 39, but there have been no previous reports on whether it could predict the efficacy of antidepressants. However, in a recent study involving patients with depressive disorders, including MDD and dysthymic disorder, it was observed that baseline leptin levels were significantly elevated in the non-remission group compared to the remission group. These findings imply a connection between Leptin R and the therapeutic effects of antidepressants, yet the precise mechanism remains to be further explored. Future research may need to investigate how Leptin R, along with other factors, can predict the therapeutic effects of antidepressants.

Our research suggests that cytokines are superior to peripheral immune cells in predicting the efficacy of antidepressant treatment. Furthermore, we have found that significantly altered cytokines in depressive disorders can be classified into four categories based on their dynamic changes post-treatment. Notably, three of these categories tend to revert to normal levels, suggesting that cytokines may serve as viable targets for antidepressant therapy. Several of these factors, including bFGF and VEGFR1 40,41 have been previously reported to have associations with antidepressant effects. Additionally, we identified new indicators such as CXCL14, which showed significant increases in patients with MDD and considerable decreases following an eight-week treatment period.

This study has several limitations. First, as a single-center cohort study conducted at the Shanghai Mental Health Center, it included only Chinese patients, which may introduce potential selection bias. Second, our sample size remains relatively small and does not meet the 10 events per predictor variable (EPV) rule. Specifically, there is an imbalance in sample sizes between MDD patients (n = 134) and healthy controls (n = 50), which may affect the model's performance and its generalizability. However, despite these limitations, this exploratory study represents, to the best of our knowledge, the largest sample size utilizing CyTOF and Quantibody array technology to detect immune cells and cytokines in MDD research. Our model demonstrates robust performance with high AUC, accuracy, recall, and F1-Score metrics in both the diagnostic model's test set and the bootstrap validation set for treatment prediction, showing no substantial decline compared to the combined development set of both models. This may suggest that the models are not substantially affected by potential overfitting associated with not meeting the 10 EPV rule. Finally, our study lacks validation from an independent external cohort. Although we employed internal validation strategies to demonstrate the diagnostic and therapeutic predictive efficiency of the model, additional prospective and independent studies are necessary to validate our findings and models across multiple centers and cohorts of different ethnicities.

In conclusion, we have constructed a diagnostic model featuring 16 indicators derived from CyTOF and cytokines, demonstrating high diagnostic value. Contrary to the approach taken in constructing the diagnostic model, we found that building a model to predict antidepressant treatment response based solely on immune cells might not yield optimal results. In contrast, leveraging cytokines for such predictions proved valuable, leading us to construct a seven-factor treatment response prediction model. Importantly, we noted that most significantly altered cytokines in MDD can revert to normal levels following antidepressant treatment, suggesting that these cytokines could serve as potential targets for antidepressant therapy.

## Supplementary Material

Supplementary figures and tables.
