# Supplementary material for: Development of machine-learning-driven signatures for diagnosing and monitoring therapeutic response in major depressive disorder using integrated immune cell profiles and plasma cytokines
Source: Theranostics. 2024 Oct 28;14(18):7265–80. doi: 10.7150/thno.102602 (PMC11610142; doi:10.7150/thno.102602)
Supplement: Supplementary file 1 — Supplementary figures and tables. [file thnov14p7265s1.zip › Supplementary Figures and Tables.pdf]

Supplementary Figures and Tables

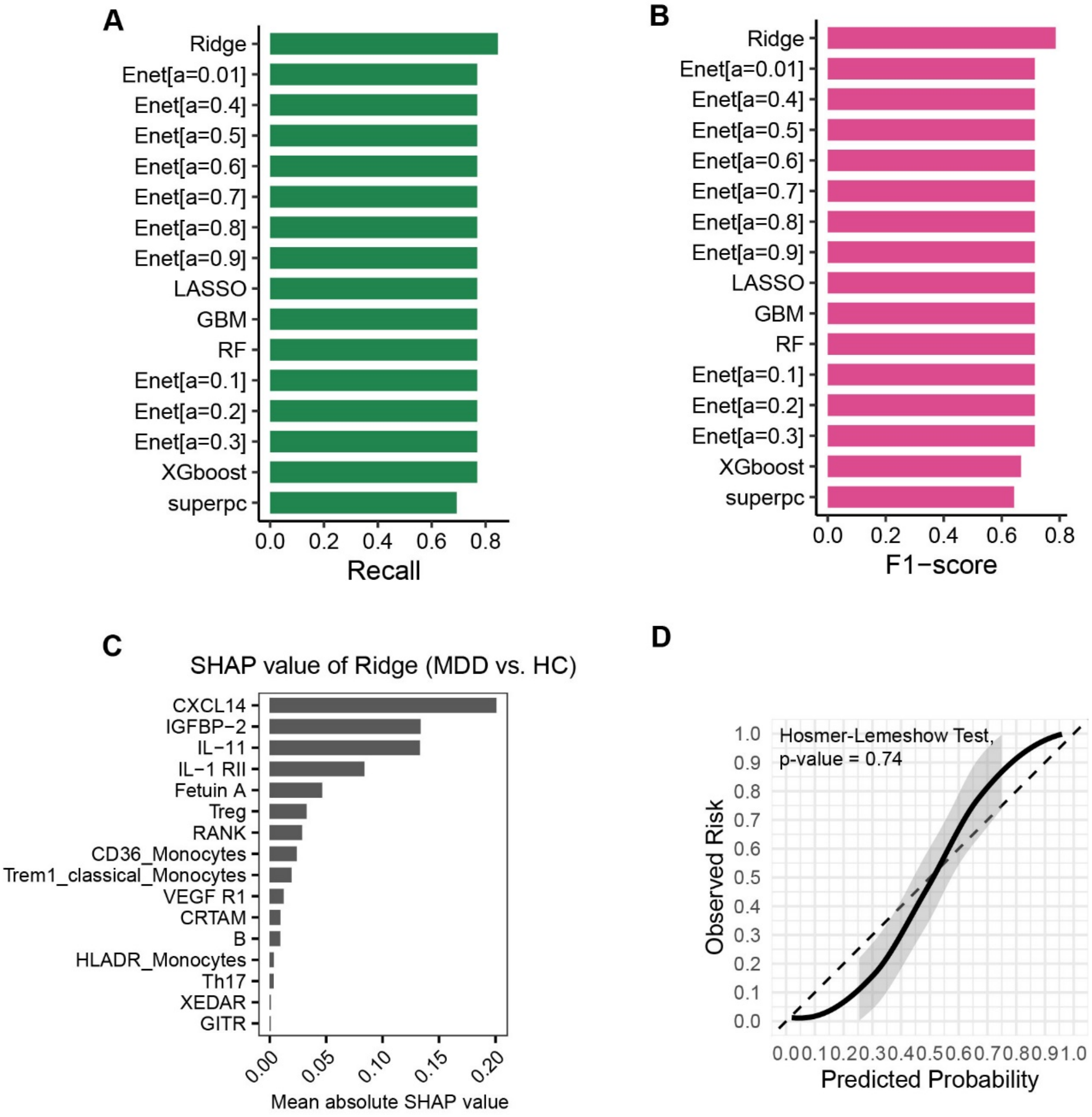

Figure S1. Evaluation and visualization of machine learning algorithms for Major Depressive Disorder (MDD) diagnostic model performance.

(A-B) High recall and F1-score values achieved by eight algorithms, with ridge regression exhibiting the highest values simultaneously. (C) The SHAP values of ridge regression for the MDD diagnostic model. (D) Calibration curve of the MDD diagnosis model based on ridge regression algorithm.

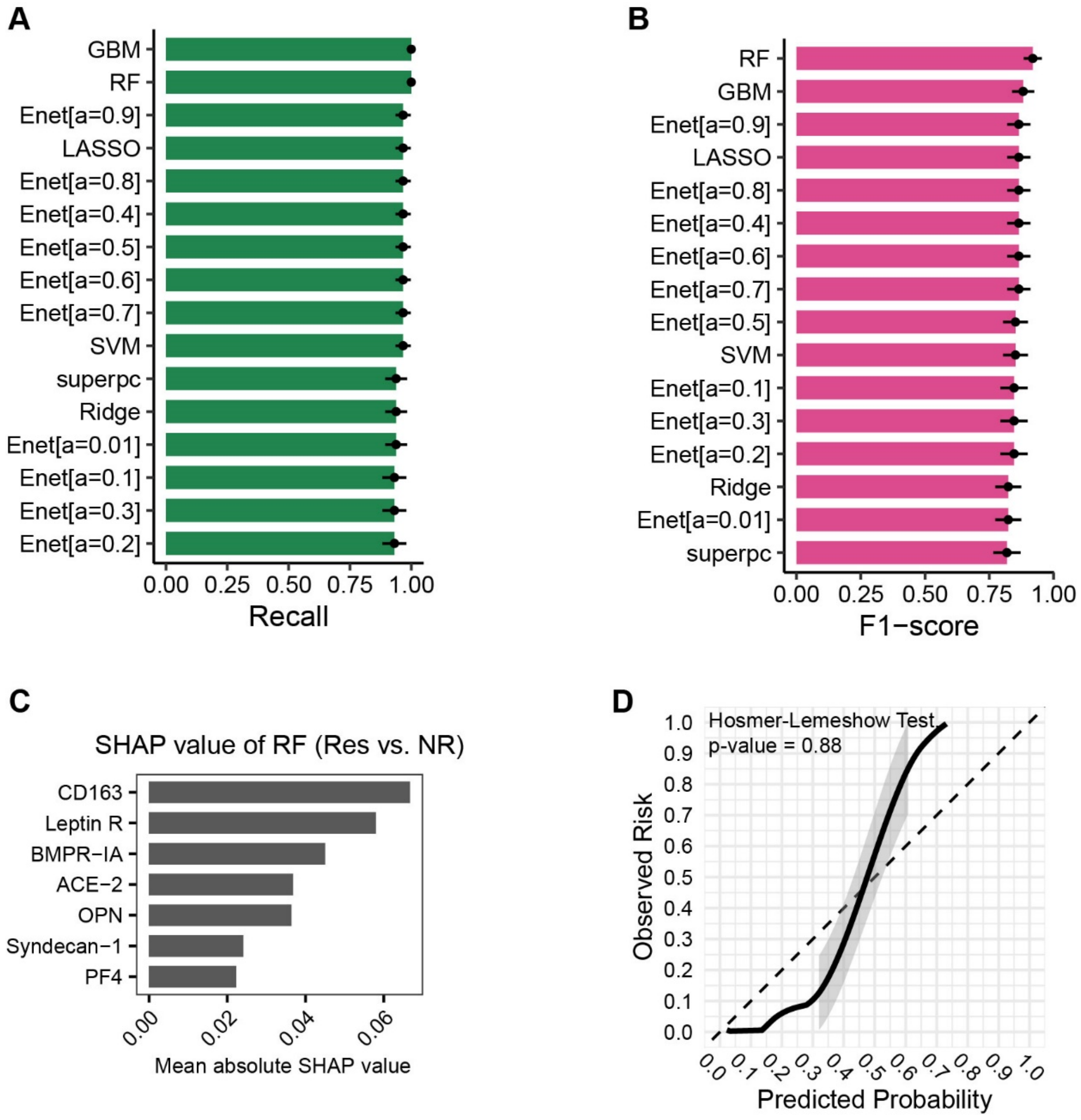

Figure S2. Evaluation and visualization of machine learning algorithms for Major Depressive Disorder (MDD) treatment response prediction model.

(A-B) High recall and F1-score values achieved by eight algorithms for the MDD treatment response prediction model. (C) The SHAP values of Random Forest (RF) for the MDD treatment response prediction model. (D) Calibration curve of the MDD treatment response prediction model based on the RF algorithm.

Supplementary Table 1. Demographic and clinical characteristics of MDD patients and controls who completed CyTOF detection

	No. (%)			
Characteristic	MDD group (n=134)	Control group (n=50)	Statistical test result	*P* value
Age, mean (SD), y	27.1(7.4)	26.4(9.2)	-1.85[a]	0.06
Sex				
Female	98(73.1)	35(70.0)	0.18[b]	0.67
Male	36(26.9)	15(30.0)		
BMI, mean (SD)	22.7(4.4)	22.7(3.5)	-0.15[a]	0.88
MADRS total score, mean (SD)	32.4(5.5)	1.4 (1.8)	-10.45[a]	<0.001
HAMD total score, mean (SD)	25.2(4.2)	1.4(1.6)	-10.45[a]	<0.001
Family history	22(16.4)	1(2.0)	6.92[b]	0.009
Length of current episode, mean (SD), month	8.3(10.5)			
Duration of MDD, mean (SD), month	25.5(38.1)			
Medication history				
Drug naïve or drug free	99(73.9)			
Pre-enrollment SSRIs usage	24(17.9)			
Pre-enrollment SNRIs usage	7(5.2)			
Pre-enrollment usage of other types of antidepressants	4(3.0)			

Abbreviations: MDD, major depressive disorder; CyTOF, Cytometry by Time-Of-Flight; BMI, body mass index (calculated as weight in kilograms divided by height in meters squared); MADRS, Montgomery–Åsberg Depression Rating Scale; HAMD, Hamilton Rating Scale for Depression; SSRIs, selective serotonin reuptake inhibitors; SNRIs, serotonin and norepinephrine reuptake inhibitors.

[a] Mann-Whitney U Test

[b] Chi-Square Test

Supplementary Table 2. Demographic and clinical characteristics of MDD patients and controls who completed cytokine detection.

Characteristic	No. (%) MDD group (n=84)	Control group (n=50)	Statistical test result	*P* value
Age, mean (SD), y	26.6(7.9)	26.4(9.2)	-1.10[a]	0.27
Sex				
Female	59(70.2)	35(70.0)	0.001[b]	0.98
Male	25(29.8)	15(30.0)		
BMI, mean (SD)	22.8(4.6)	22.7(3.5)	-0.009[a]	0.99
Family history	13(15.7)	1(2.0)	6.18[b]	0.01
MADRS total score, mean (SD)	32.3(5.2)	1.4(1.8)	-9.70[a]	<0.001
HAMD total score, mean (SD)	25.5(4.0)	1.4(1.6)	-9.69[a]	<0.001
Duration of current episode, mean (SD), mo	7.7(9.7)			
Duration of MDD, mean (SD), mo	20.1(35.6)			
Medication history				
Drug naïve or drug free	66(78.6)			
Pre-enrollment SSRIs usage	13(15.5)			
Pre-enrollment SNRIs usage	3(3.6)			
Pre-enrollment usage of other types of antidepressants	2(2.4)			

Abbreviations: MDD, major depressive disorder; BMI, body mass index (calculated as weight in kilograms divided by height in meters squared); MADRS, Montgomery–Åsberg Depression Rating Scale; HAMD, Hamilton Rating Scale for Depression; SSRIs, selective serotonin reuptake inhibitors; SNRIs, serotonin and norepinephrine reuptake inhibitors.

[a] Mann-Whitney U Test

[b] Chi-Square Test

Supplementary Table 5. Demographic and Clinical Characteristics of Responder and Nonresponder Patients who Completed CyTOF Detection.

Characteristic	No. (%) Responder (n=56)	Nonresponder (n=28)	Statistical test result	*P* value
Age, mean (SD), y	27.1(7.0)	27.6(9.3)	0.00[a]	>0.999
Sex				
Female	37(66.1)	22(78.6)	1.40[b]	0.24
Male	19(33.9)	6(21.4)		
BMI, mean (SD)	22.9(3.7)	22.7(4.3)	-0.22[a]	0.82
Family history	9(16.1)	9(32.1)	2.86 [b]	0.09
MADRS total score, mean (SD)				
Week-0	32.2(5.0)	32.1(5.0)	-0.05[c]	0.96
Week-8	8.0(4.7)	21.3(4.5)	-7.31[a]	<0.001
HAMD total score, mean (SD)				
Week-0	24.2(4.2)	25.3(4.0)	-1.07[a]	0.29
Week-8	7.1(4.5)	15.3(4.6)	-5.84[a]	<0.001
Duration of current episode, mean (SD), mo	8.3(11.0)	11.8(11.0)	-1.85[a]	0.06
Duration of MDD, mean (SD), mo	24.1(33.1)	39.0(47.1)	-1.88[a]	0.06
Medication during 8 weeks				
SSRIs during 8 weeks	44(78.6)	22(78.6)	0.57[d]	>0.999
SNRIs during 8 weeks	11(19.6)	6(21.4)		
Usage of other types of antidepressants or combination of two antidepressant medications	1(1.8)	0(0.0)		

Abbreviations: CyTOF, Cytometry by Time-Of-Flight; BMI, body mass index (calculated as weight in kilograms divided by height in meters squared); MADRS, Montgomery–Åsberg Depression Rating Scale; HAMD, Hamilton Rating Scale for Depression; SSRIs, selective serotonin reuptake inhibitors; SNRIs, serotonin and norepinephrine reuptake inhibitors.

[a] Mann-Whitney U Test

[b] Chi-Square Test

[c] Student's t test

[d] Fisher's exact test

Supplementary Table 6. Demographic and Clinical Characteristics of Responder and Nonresponder Patients who Completed cytokine Detection.

Characteristic	No. (%) Responder (n=31)	Nonresponder (n=18)	Statistical test result	*P* value
Age, mean (SD), y	25.4(6.3)	27.9(11.0)	-0.54[a]	0.59
Sex				
Female	20(64.5)	12(66.7)	0.02[b]	0.88
Male	11(35.5)	6(13.3)		
BMI, mean (SD),	23.1(3.5)	22.8(5.0)	-0.60[a]	0.55
Family history	4(12.9)	5(27.8)	0.84[c]	0.36
MADRS total score, mean (SD)				
Week-0	32.0(4.8)	31.8(3.3)	-0.17[d]	0.87
Week-8	7.3(4.7)	20.1(3.4)	-5.75[a]	<0.001
HAMD total score, mean (SD)				
Week-0	24.7(4.6)	25.7(3.3)	-0.89[a]	0.38
Week-8	7.0(4.9)	14.0(4.2)	-4.04[a]	<0.001
Duration of current episode, mean (SD), mo	7.8(8.4)	10.6(9.7)	-1.32[a]	0.19
Duration of MDD, mean (SD), mo	12.8(14.8)	32.8(47.1)	-1.70[a]	0.09
Medication during 8 weeks				
SSRIs during 8 weeks	24(77.4)	13(72.2)	1.02[e]	0.83[b]
SNRIs during 8 weeks	6(19.4)	5(27.8)		
Usage of other types of antidepressants or combination of two antidepressant medications	1(3.2)	0(0.0)		

Abbreviations: BMI, body mass index (calculated as weight in kilograms divided by height in meters squared); MADRS, Montgomery–Åsberg Depression Rating Scale; HAMD, Hamilton Rating Scale for Depression; SSRIs, selective serotonin reuptake inhibitors; SNRIs, serotonin and norepinephrine reuptake inhibitors.

[a] Mann-Whitney U Test

[b] Chi-Square Test

[c] Yates's correction for continuity

[d] Student's t test

[e] Fisher's exact test
